# Impact of a continuing medical education meeting on the use and timing of urgent cancer referrals among general practitioners - a before-after study

**DOI:** 10.1186/s12875-017-0607-3

**Published:** 2017-03-21

**Authors:** Berit Skjødeberg Toftegaard, Flemming Bro, Alina Zalounina Falborg, Peter Vedsted

**Affiliations:** 1 Research Unit for General Practice, Department of Public Health, Aarhus University, Bartholins Allé 2, Aarhus, DK-8000 Denmark; 2 Research Centre for Cancer Diagnosis in Primary Care (CaP), Department of Public Health, Aarhus University, Bartholins Allé 2, Aarhus, DK-8000 Denmark; 3 Section for General Medical Practice, Department of Public Health, Aarhus University, Bartholins Allé 2, Aarhus, DK-8000 Denmark; 4 Department of Clinical Medicine, University Clinic for Innovative Health Care Delivery, Silkeborg Hospital, Aarhus University, Aarhus, DK-8000 Denmark

**Keywords:** Denmark, General practice, Continuing medical education meeting, Diagnosis, Early detection of cancer, Behavioural change, Referral rate, Primary care interval, Use of health care

## Abstract

**Background:**

Detection of cancer in general practice is challenging because symptoms are diverse. Even so-called alarm symptoms have low positive predictive values of cancer. Nevertheless, appropriate referral is crucial. As 85% of cancer patients initiate their cancer diagnostic pathway in general practice, a Continuing Medical Education meeting (CME-M) in early cancer diagnosis was launched in Denmark in 2012. We aimed to investigate the effect of the CME-M on the primary care interval, patient contacts with general practice and use of urgent cancer referrals.

**Methods:**

A before-after study was conducted in the Central Denmark Region included 396 general practices, which were assigned to one of eight geographical clusters. Practices were invited to participate in the CME-M with three-week intervals between clusters. Based on register data, we calculated urgent referral rates and patient contacts with general practice before referral. Information about primary care intervals was collected by requesting general practitioners to complete a one-page form for each urgent referral during an 8-month period around the time of the CME-Ms. CME-M practices were compared with non-participating reference practices by analysing before-after differences.

**Results:**

Forty percent of all practices participated in the CME-M. There was a statistically significant reduction in the number of total contacts with general practice from urgently referred patients in the month preceding the referral and an increase in the proportion of patients who waited 14 days or more in general practice from the reported date of symptom presentation to the referral date from before to after the CME-M in the CME-M group compared to the reference group.

**Conclusions:**

We found a reduced number of total patient contacts with general practice within the month preceding an urgent referral and an increase in the reported primary care intervals of urgently referred patients in the CME-M group. The trend towards higher urgent referral rates and longer primary care intervals may suggest raised awareness of unspecific cancer symptoms, which could cause the GP to register an earlier date of first symptom presentation. The standardised CME-M may contribute to optimising the timing and the use of urgent cancer referral.

**Trial registration:**

NCT02069470 on ClinicalTrials.gov. Retrospectively registered, 1/29/2014

**Electronic supplementary material:**

The online version of this article (doi:10.1186/s12875-017-0607-3) contains supplementary material, which is available to authorized users.

## Background

Danish cancer patients have lower survival and more advanced disease stages at treatment initiation than many other European countries [1, 2]. The ability among general practitioners (GPs) to interpret and respond to symptoms plays a pivotal role in cancer detection as 85% of all cancer patients initiate their diagnostic pathway in general practice [3, 4]. Increasing evidence suggests that prompt referral from general practice matters [5–7], and a UK study found that a high propensity to use urgent referrals was associated with better survival of cancer patients [8].

Cancer detection in general practice is, however, a challenging task. The symptoms of cancer are diverse, they develop over time, and they tend to mimic symptoms of trivial and common conditions [9]. Some symptoms are unspecific, e.g. fatigue and weight loss. Others are considered to be alarm symptoms [10] although they have a low positive predictive value of cancer of only 2-10%, depending on age, gender and cancer type [11–13]. Danish cancer patients have increased visits in general practice already six months before the diagnosis [14], and one in four waits for more than 20 days in general practice until referral [4, 15]. Consequently, there may be room for improvements.

To support the GPs’ decision strategies for referral, a specific Continuing Medical Education meeting (CME-M) was developed and implemented as part of the Danish National Cancer Plan III in 2012 [16]. The developed CME-M consisted of central elements for cancer detection in primary care [17]. So far, the initial evaluation has shown that the CME-M has affected the knowledge among GPs on cancer diagnosis and their attitude towards own role in cancer detection. The CME-M also lowered the GPs’ assessed risk of cancer in urgently referred patients [18]. Yet, we need to explore whether the change in GPs’ knowledge and attitude was translated into a behavioural change.

The aim of this study was to investigate the effect of the CME-M on the use of urgent cancer referrals by investigating urgent referral rates and the timing of urgent referrals in terms of length of primary care interval and number of patient contacts with general practice before referral.

## Methods

The setting of the study was the Central Denmark Region with approx. 1.3 million inhabitants and 8,000 new cancer patients annually [19]. The Danish healthcare system is tax-funded with free access for citizens to medical advice and treatment in general practice and hospitals. More than 98% of the Danish citizens are listed with a specific general practice, which they must consult for medical advice. GPs serve as gatekeepers to specialised care, such as diagnostic investigations and hospital care [20]. In order to ensure consistency in the GPs’ selection of patients for urgent cancer referrals, national referral guidelines were made for each cancer fast-track pathway [21, 22]. As an example, the referral guideline for colorectal cancer state that cancer should be considered if a patient above 40 years of age presents at least one of the following symptoms: visible rectal bleeding, discharge of mucus or changes in bowel habits or stools for a four-week period, iron-deficiency anaemia and unspecific symptoms as pain or weight loss [21]. All Danish citizens have a civil personal registration (CPR) number, which enables linkage of information at the individual level between national registries and allows identification of the practice at which each citizen is listed [23].

### Design

**Fig. 1:**
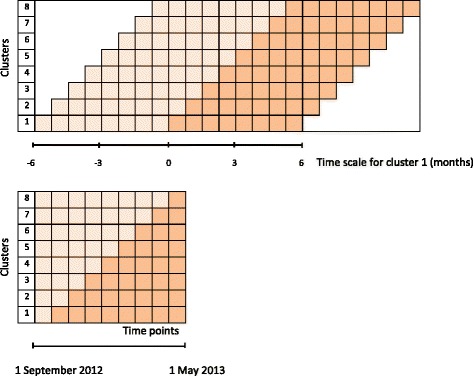
Time periods used for classifying patient populations. The cluster-specific time period used for identifying urgently referred patients in the primary care referral database (l*ight boxes* = time before CME-M; *dark boxes* = time after CME-M). The method at the top was applied for evaluating CME-M effect on referral rates and patient contacts with general practice. The method at the bottom was used for evaluating CME-M effect on primary care intervals

We conducted a before-after study with a stepwise enrolment of GPs in the CME-Ms [17]. All practices in the Central Denmark Region were allocated to one of eight clusters by exploiting the already existing municipal units. The Regional Cancer Quality Unit assigned the random order in which all GPs (practices) in the cluster were offered the CME-M at 3-week intervals within the period 1 September 2012 to 1 May 2013 (Fig. 1). GPs were registered if they participated in the CME-M [17].

### Intervention: continuing medical education meeting

The content of the CME-M was developed through a comprehensive process, which has been described previously in detail [17]. The CME-M provided the GPs with new knowledge on symptoms of cancer and presented risk assessment tools (RATs) for lung, colorectal, ovarian and prostate cancer [13, 24]. Use of investigations and reasons for missed opportunity [25, 26] were discussed in terms of risk of false reassurance [27, 28]. Mechanisms affecting referral thresholds were debated based on patient cases [29].

### Outcomes

Use of urgent referrals was operationalised as: 1) *urgent referral rate per GP* (i.e. number of urgently referred patients during six months per GP in the practice) and 2) *urgent referral rate per 1000 patients in the practice* (i.e. number of urgently referred patients during six months per 1000 listed patients aged ≥ 40 years in the practice). Timing of urgent referrals was operationalised as: 1) *prior contacts* (i.e. number of daytime contacts with general practice from urgently referred patients before referral) and 2) *primary care interval* (i.e. number of days from first presentation in general practice until urgent referral to secondary care) [30].

### Practice population

The practices were identified in the Danish provider number registry. The number of included practices depended on the outcome to be measured. For *urgent referral rates,* practices were included if registered from six months before to six months after their CME-M date. For prior *contacts,* practices were included if registered from 18 months before to six months after their CME-M date. For *primary care interval*, practices were included if registered in the period from 1 September 2012 to 1 May 2013 provided that they had completed one-page forms (Additional file 1).

### Patient populations

The urgently referred patients were identified by applying an algorithm, which was developed for the purpose, to the primary care referral database [31]. The algorithm had a sensitivity of 93.6%, a specificity of 97.3%, a positive predictive value of 83.6% and a negative predictive value of 99.0% for identifying urgently referred patients aged ≥ 40 years without prior cancer among all referred patients aged ≥ 40 years [31].

For *urgent referral rates*, patients were included if: 1) identified by the algorithm, 2) referred from an included practice from six months before until six months after the CME-M date of the practice, 3) age ≥ 40 years and 4) no prior cancer.

For *prior contacts,* patients were included on the same criteria as above. Furthermore, they were also required to have been listed with the same practice for one year prior to the referral date.

For *primary care interval*, patients were included if: 1) identified by the algorithm, 2) referred from an included practice from 1 September 2012 to 1 May 2013, 3) age ≥ 40 years, 4) no prior cancer and 5) receipt of one-page form stating the date of first symptom presentation and the date of referral.

Patients already diagnosed with cancer were excluded based on data in the Danish Cancer Registry [32] registered in accordance with the International Classification of Diseases, 10^th^ revision: C0-C9, except for non-melanoma skin cancer (C44) [33].

### Variables

Patient contacts with general practice were obtained from the Danish National Health Service Register [34]. The registration is based on fee-for-service remuneration of the providing GP, and the records in this registry are generally considered highly complete [35]. We used two variables: 1) face-to-face (F2F) contacts and 2) total contacts, which included F2F, phone and e-mail contacts.

Date of first presentation of symptom in general practice and date of referral were collected by requesting GPs to complete a one-page form including the patient’s CPR number each time a patient was referred for suspected cancer during an 8-month period around the CME-Ms (1 September 2012 to 1 May 2013) (Fig. 1) [17]. Furthermore, in the hospital patient administrative system, patients registered as investigated as part of an urgent referral were identified every other week. If the one-page form was missing, the relevant GP was requested to complete a form and to specify the patient’s route to investigation. This was done to increase the completeness of data for urgently referred patients.

The development, pilot-testing, coding and data transfer of the forms have been reported elsewhere [17].

Age and gender distribution of the patient population for each practice was obtained from the patient list system on 1 January 2013.

The Danish Deprivation Index (DADI), which is used to adjust for differences in socioeconomic factors, was calculated based on data from the Integrated Database for Labour Market Research [36]. The DADI has a value between 10 and 100; the higher the number, the more deprived population. The variables used were: (i) proportion of adults aged 20–59 years with no employment, (ii) proportion of adults aged 25–59 years with no professional education, (iii) proportion of adults aged 25–59 years with low income, (iv) proportion of adults aged 18–59 years receiving public welfare benefits (transfer payments or social security benefits), (v) proportion of children from parents with no education and no professional skills, (vi) proportion of immigrants, (vii) proportion of adults aged 30+ years living alone and (viii) proportion of adults aged 70+ years with low income (= the lowest national quartile).

A modified Charlson Comorbidity Index score [37] was obtained for each referred patient by using data from the Danish National Patient Register with the referral date as the index date [38]. We divided the comorbidity scores into “none” (no recorded disease), “moderate” (score of 1 or 2) and “high” (score of 3 or more) [15].

The suspected cancer type stated at the one-page form was divided into two groups of cancer detection difficulty based on the available evidence [39]. Cancers considered e*asier to detect* included suspected cancer in kidney, bladder, breast, head and neck, female genitalia, nevus (melanoma), penis, testis and gastrointestinal system, whereas cancers considered *harder to detect* included suspected cancer in pancreas, liver and gall bladder, brain, lymph node and bone marrow, lung, prostate, connective tissue including fat, muscle and bones, and suspicion based on unspecific serious symptoms.

### Statistical analyses

Practices were divided into two groups: practices with at least one CME-M-participating GP (“CME-M practices”) and practices without any CME-M-participating GPs (“reference practices”).

The time point for a CME-M session for each cluster was defined as the point that separated the time of observation in “before CME-M” and “after CME-M” (Fig. 1). The data collected before and after the CME-M provided paired responses for each outcome. The practice and patient populations were described by their characteristics.

*Urgent referral rates* were indirectly age-sex standardised as we used the full patient population in the Central Denmark Region as standard population divided into ten-year age groups (40–49, 50–59, etc.).

The CME-M effect on *urgent referral rates* was analysed using mixed-effects negative binomial regression [40] with a random effect associated with the intercept for each practice to make comparisons before and after CME-M within and between groups. The model allowed dealing with repeated observations on practice level. The time of observation (before and after CME-M) was treated as a fixed-effect variable. In the analysis of *urgent referral rates per GP*, the number of GPs per practice was treated as an exposure variable. The effects within groups were reported as incidence rate ratios (IRRs). Comparison of effects between groups was reported as a ratio of the IRRs. The analyses were adjusted for cluster, type of practice (single-handed/partnership) and DADI index. A single-handed practice was defined as a practice with only one GP. A stratified analysis was performed on practice type.

*Prior contacts* were measured in different time intervals: 0–1 month, 0–3 months and 4–6 months preceding referral. The number of contacts during the 7–12 months preceding referral was calculated to estimate an average habitual contact rate per month for each patient.

The CME-M effect on *prior contacts* was analysed using mixed-effects negative binomial regression with a random effect associated with the intercept for each practice and time of observation as a fixed-effect variable to make comparisons before and after CME-M within and between groups while adjusting for patient clustering in the practices. The results were reported as IRRs or a ratio of the IRRs. Due to convergence problems for F2F within the last month preceding the referral, the analysis was based on ordinary negative binomial regression applying cluster robust variance at practice level.

For the 10% of patients with most frequent contact, the number of F2F contacts and total contacts were dichotomised based on the 90^th^ percentile (number of contacts within 0–1 month was 4 for F2F and 5 for total contacts). A mixed-effects logistic regression [41] allowed for random effects at practice level, and the observation time (before and after) was treated as a fixed-effect variable. The effects were reported as odds ratios (ORs) for having most contacts within groups and ratio of the ORs between groups.

The analyses were adjusted for cluster, practice type, patient gender, age, habitual monthly contact rate and comorbidity. Furthermore, a stratified analysis on practice type was performed.

The *primary care intervals* were calculated as medians, 75^th^ and 90^th^ percentiles. We dichotomised the intervals based on the 75^th^ and 90^th^ percentiles from before the CME-M. Comparisons within and between groups were estimated with mixed-effects logistic regression with a random effect associated with the intercept for each practice adjusting for patient clustering in the practices. The results were reported as ORs or a ratio of the ORs. The analyses were adjusted for cluster, practice type, patient gender, age, co-morbidity and cancer detection difficulty. Furthermore, stratified analyses were performed on cancer detection difficulty and type of practice.

The statistical software Stata 13.0 (StataCorp LP, TX, USA) was used for the analyses [42].

## Results

### Study population

A total of 148 of the 396 general practices (37.4%) participated in the CME-M (Table 1). Compared to the reference practices, the CME-M practices were more often partnership practices, the GPs were slightly younger and more often female, the practice population size per GP was lower, the practice population consisted of fewer male and slightly less deprived patients (Tables 1 and 2).

**Table 1:** Characteristics of practices, GPs and patients used for CME-M impact on urgent referral rates

	Reference group		CME-M group	
Study base, Practices, *N*	248		148	
Proportion of solo practices (95% CI)	0.60 (0.54;0.66)		0.30 (0.23;0.38)	
GPs per practice, mean (range)	1.72 (1–5)		2.60 (1–10)	
GPs, *N*	*425*		*385*	
Proportion of males (95% CI)	0.59 (0.54;0.64)		0.48 (0.43;0.53)	
Mean age (95% CI)	52.6 (51.7;53.4)		51.6 (50.8;52.4)	
Underlying practice populations, *N*	661,805		570,926	
Mean list size, patients ≥ 40 yrs. per practice	1353		1920	
Mean list size, patients ≥ 40 yrs. per GP in practice	787		738	
Proportion of male patients aged ≥ 40 yrs. (95% CI)	0.50 (0.49;0.50)		0.48 (0.47;0.49)	
Age groups (yrs.), proportion of all patients ((%)(95% CI))				
0–39	48.6 (47.4; 49.9)		49.9 (48.4; 51.3)	
40–49	14.1 (13.8;14.4)		14.0 (13.7;14.4)	
50–59	13.4 (13.0;13.7)		12.7 (12.2;13.1)	
60–69	12.5 (12.0;12.9)		12.1 (11.6;12.6)	
70–79	7.3 (6.9;7.6)		7.2 (6.8;7.6)	
*80–90*	3.5 (3.3; 3.7)		3.4 (3.2; 3.6)	
Above 90	0.76 (0.7; 0.8)		0.73 (0.7; 0.8)	
Mean DADI index (95% CI)	26.9 (25.9;27.8)		25.5 (24.4;26.5)	
Referred patients (age ≥ 40 yrs.)	*Before CME-M, N = 8,388*	*After CME-M,* *N = 8,534*	*Before CME-M,* *N = 7,579*	*After CME-M,* *N = 7,942*
Proportion of males (95% CI)	0.47 (0.45;0.48)	0.45 (0.44;0.46)	0.44 (0.43;0.46)	0.44 (0.43;0.45)
Mean age (95% CI)	63.0 (62.7;63.3)	63.1 (62.9;63.4)	62.6 (62.3;62.9)	62.8 (62.5;63.1)
Comorbidity^a^ (%)				
None	75.5	74.8	76.2	75.2
Medium	20.9	21.3	19.9	21.0
High	3.6	3.9	3.9	3.8

*Abbreviations:* *CME-M* continuing medical education meeting, *GP* general practitioner, *CI* confidence interval; yrs., years, *DADI* Danish Deprivation Index

^a^Comorbidity scores were divided into “none” (no recorded disease),“ moderate” (score of 1 or 2) and “high” (score of 3 or more)

**Table 2:** Characteristics of practices, GPs and patients used for CME-M impact on patients’ prior contacts

	Reference group		CME-M group	
Practices, *N*	240		144	
Proportion of solo practices (95% CI)	0.60 (0.54;0.66)		0.29 (0.22;0.37)	
GPs per practice, mean (range)	1.73 (1–5)		2.63 (1–10)	
GPs, *N*	415		377	
Proportion of males (95% CI)	0.60 (0.55;0.64)		0.48 (0.43;0.53)	
Mean age (95% CI)	52.9 (52.0;53.7)		51.7 (50.9;52.5)	
Referred patients (age ≥ 40 yrs.), *N*	*Before CME-M, N = 7,659*	*After CME-M, N = 7,731*	*Before CME-M, N = 7,121*	*After CME-M, N = 7,417*
Proportion of males (95% CI)	0.46 (0.45;0.47)	0.46 (0.45;0.47)	0.44 (0.43;0.46)	0.44 (0.43;0.45)
Mean age (95% CI)	63.1 (62.8;63.4)	63.2 (62.9;63.5)	62.7 (62.4;63.0)	62.9 (62.6;63.2)
Comorbidity (%)^a^				
None	75.9	75.3	76.4	75.7
Medium	20.7	21.1	19.8	20.8
High	3.4	3.6	3.8	3.6
Mean habitual F2F monthly contact rate^b^ (95% CI)	0.60 (0.58;0.61)	0.57 (0.56;0.59)	0.61 (0.59;0.62)	0.60 (0.58;0.61)
Mean habitual total monthly contact rate^b^ (95% CI)	1.05 (1.03;1.08)	1.02 (1.00;1.05)	1.07 (1.05;1.10)	1.04 (1.01;1.06)

*Abbreviations:* *CME-M* continuing medical education meeting, *GP* general practitioner, *CI* confidence interval; yrs., years, *DADI* Danish Deprivation Index, *F2F* face-to-face

^a^Comorbidity scores were divided into “none” (no recorded disease), “ moderate” (score of 1 or 2) and “high” (score of 3 or more)

^b^Mean habitual monthly contact rate: average contacts per month based on number of contacts 7–12 months preceding referral for each patient

The patients used for calculation of the *primary care interval* were included by 139 CME-M practices and 199 reference practices (Table 3).

**Table 3:** Characteristics of practices, GPs and patients used for CME-M impact on patients’ primary care interval

	Reference group		CME-M group	
Practices, *N* (proportion of study base %)	199 (82.9%)		139 (93.9%)	
Proportion of solo practices (95% CI)	0.53 (0.46;0.60)		0.26 (0.19;0.35)	
GPs per practice, mean (range)	1.85 (1–5)		2.66 (1–10)	
GPs, *N*	369		370	
Proportion of males (95% CI)	0.57 (0.52;0.62)		0.47 (0.42;0.53)	
Mean age (95% CI)	52.1 (51.2;53.0)		51.4 (50.6;52.2)	
Total referred patients during 8 months (age ≥ 40 yrs.)	*Before CME-M, N = 5,749*	*After CME-M, N = 4,607*	*Before CME-M, N = 4,935*	*After CME-M, N = 5,303*
Proportion of males (95% CI)	0.46 (0.45;0.47)	0.45 (0.44;0.47)	0.44 (0.43;0.46)	0.44 (0.43;0.46)
Mean age (95% CI)	62.8 (62.5;63.1)	63.0 (62.6;63.4)	62.5 (62.1;62.8)	62.8 (62.5;63.2)
Comorbidity (%)				
None	75.3	74.7	75.8	74.8
Medium	20.7	21.3	19.9	21.2
High	4.0	4.0	4.3	3.9
Referred patients with known primary care intervals (age ≥ 40 yrs.) (Response (%))	*Before CME-M, N = 1,227* *(21.3%)*	*After CME-M, N = 753* *(16.3%)*	*Before CME-M, N = 990* *(20.0%)*	*After CME-M, N = 1,102* *(20.8%)*
Proportion of males (95% CI)	0.52 (0.49;0.55)	0.54 (0.50;0.57)	0.48 (0.45;0.51)	0.47 (0.44;0.50)
Mean age (95% CI)	64.9 (64.2;65.6)	64.5 (63.6;65.3)	64.5 (63.8;65.3)	64.8 (64.1;65.5)
Comorbidity^a^ (%)				
None	76.7	75.4	76.1	75.6
Medium	19.7	20.2	20.1	21.0
High	3.6	4.4	3.8	3.4
Cancer detection difficulty^b^ (%)				
Easier to detect	72.2	68.4	69.2	70.2
Harder to detect	23.1	25.4	26.4	24.9
Missing information	4.6	6.2	4.4	4.9

*Abbreviations:* *CME-M* continuing medical education meeting, *GP* general practitioner, *CI* confidence interval; yrs., years

^a^Comorbidity scores were divided into “none” (no recorded disease), “ moderate” (score of 1 or 2) and “high” (score of 3 or more)

^b^The suspected cancer was divided based on cancer detection difficulty: 1) easier to detect included kidney, bladder, breast, head and neck, female genitalia, nevus (melanoma), penis, testis and gastrointestinal and 2) harder to detect included unspecific symptoms, pancreas, liver and gall bladder, brain, lymph node and bone marrow, lung, prostate and connective tissue, including fat, muscle and bones

### Impact of CME-M on urgent referral rates

The CME-M was associated with a two-fold relative rise in *urgent referral rates per GP* when comparing CME-M practices with reference practices. Within the CME-M group, this increase was statistically significant (IRR: 1.05 (95% CI: 1.01;1.08) (Table 4). Each CME-M practice referred one patient more per 1000 patients in their underlying practice population during the 6-month period after the CME-M compared to before; this increase was three times higher than the increase in reference practices (Table 4). When we stratified on practice type, the partnership practices who participated in the CME-M increased their *urgent referral rate per GP* (IRR: 1.06 (95% CI: 1.02;1.10) and their *urgent referral rate per 1000 patients* (IRR: 1.06 (95% CI: 1.01;1.12) from before to after the CME-M.

**Table 4:** The CME-M impact on urgent referral rates

	Reference group					CME-M group					Comparison between groups		
	Before CME-M	After CME-M	Before vs. after			Before CME-M	After CME-M	Before vs. after					
			∆0	IRR0^b^ (95% CI)	P			∆1	IRR1^b^ (95% CI)	P	∆1/∆0	IRR1/IRR0^b^ (95% CI)	P
All data, N practices (GPs)	248 (425)		-			148 (385)		-	-		-		-
Referral rate^a^ per GP, mean	20.4	20.8	0.4	1.02^c^ (0.98;1.05)	0.316	20.6	21.4	0.8	**1.05** ^c^ **(1.01;1.08)**	**0.009**	2.0	1.03^c^ (0.98;1.08)	0.214
Referral rate^a^ per 1000 patients, mean	25.0	25.3	0.3	1.01^c^ (0.98;1.05)	0.435	27.1	28.1	1.0	1.04^c^ (0.99;1.08)	0.111	3.3	1.02^c^ (0.97;1.08)	0.454
Single-handed practices, N practices (GPs)	149 (149)		-			45 (45)		-	-		-		-
Referral rate^a^ per GP, mean	21.3	21.7	0.4	1.02 (0.96;1.08)	0.481	24.0	23.9	−0.1	0.99 (0.89;1.09)	0.796	−0.25	0.97 (0.86;1.08)	0.566
Referral rate^a^ per 1000 patients, mean	24.2	24.3	0.1	1.01 (0.95;1.07)	0.763	26.9	26.2	−0.7	0.98 (0.88;1.08)	0.626	−7.0	0.97 (0.86;1.09)	0.567
Partnership practices, N practices (GPs)	99 (276)		-			103 (340)		-	-		-		-
Referral rate^a^ per GP, mean	18.9	19.3	0.4	1.02 (0.97;1.05)	0.540	19.0	20.2	1.2	**1.06 (1.02;1.10)**	**0.002**	3.0	1.04 (0.99;1.10)	0.105
Referral rate^a^ per 1000 patients, mean	26.3	26.9	0.6	1.02 (0.97;1.08)	0.457	27.2	28.9	1.7	**1.06 (1.01;1.12)**	**0.019**	2.8	1.04 (0.97;1.12)	0.280

Indirectly age-sex standardised referral rates for 6 months before and after CME-M, respectively, are shown for 1000 patients (age ≥ 40 yrs.) per practice and per GP in the practice. Practices are divided into CME-M group and reference group. The referral rates are further stratified on practice type. The effect within a group is shown as an incidence rate ratio (IRR0: Reference group; IRR1: CME-M group). Comparisons between groups are shown as the ratio of the incidence rate ratios

*Abbreviations:* *CME-M* continuing medical education meeting, *GP* general practitioner, *IRR* incidence rate ratio, *CI* confidence interval

^a^Indirectly age-sex standardised

^b^Adjusted for cluster and Danish Deprivation index

^c^In addition, adjusted for practice type

Bold = significance level of *p* ≤ 0.05

Compared with the reference practices, none of these findings were statistically significant (Table 4). Note that the stratified analyses on practice type showed a higher use of *urgent* referrals among CME-M-participating single-handed practices compared to non-participating single-handed practices (Table 4).

### Impact of CME-M on prior contacts

There was a statistically significant reduction in the number of total contacts within the last month before referral in the CME-M group compared to the reference group (IRR-ratio: 0.97 (95% CI: 0.94;0.99)) (Table 5). Note the tendency that the proportion of patients contacting the most in terms of total contacts decreased from before to after in the CME-M group (OR-ratio: 0.90 (95% CI: 0.86;1.02)) (Table 5).

**Table 5:** The CME-M impact on patients’ prior contacts with general practice

	Reference group *N =* 240 practices				CME-M group *N =* 144 practices					
N patients	Before CME-M 7,659	After CME-M 7,731	Before vs. after		Before CME-M 7,121	After CME-M 7,417	Before vs. after		Comparison between groups	
Prior contacts			IRR0^a^ (95% CI)	P			IRR1^a^ (95% CI)	P	IRR1/IRR0^a^ (95% CI)	P
F2F contacts (mean)										
0–1 month	1.91	1.89	1.00 (0.98;1.02)	0.662	1.92	1.90	0.99 (0.97;1.01)	0.190	0.99 (0.96;1.02)	0.525
0–3 months	3.24	3.20	1.00 (0.98;1.02)	0.868	3.23	3.22	1.00 (0.98;1.02)	0.950	1.00 (0.97;1.03)	0.872
4–6 months	1.82	1.81	1.01 (0.98;1.05)	0.536	1.84	1.83	0.99 (0.96;1.03)	0.870	0.99 (0.94;1.04)	0.583
Total contacts (mean)										
0–1 month	2.84	2.87	1.01 (0.99;1.03)	0.189	2.95	2.87	**0.98 (0.94;0.99)**	**0.044**	**0.97 (0.94;0.99)**	**0.019**
0–3 months	5.17	5.21	1.01 (0.99;1.03)	0.233	5.31	5.19	0.99 (0.97;1.02)	0.369	0.98 (0.95;1.01)	0.137
4–6 months	3.22	3.24	1.00^b^ (0.97;1.04)	0.980	3.27	3.21	0.97^b^ (0.94; 1.01)	0.138	0.97^b^ (0.93;1.02)	0.296
Most contacting patients in the month preceding referral			OR0^a^ (95% CI)	P			OR1^a^ (95% CI)	P	OR1/OR0^a^ (95% CI)	P
F2F contacts ≥ 4^c^ (%)	10.4	10.4	1.02 (0.92;1.14)	0.726	10.8	10.8	0.99 (0.89;1.11)	0.911	0.97 (0.84;1.14)	0.745
Total contacts ≥ 5^c^ (%)	16.9	17.2	1.04 (0.95;1.13)	0.426	18.5	17.5	0.94 (0.86;1.02)	0.148	0.90 (0.86;1.02)	0.112

Prior contacts are shown as face-to-face and total contacts for three time periods: 0–1, 0–3 and 4–6 months and as the proportions of patients who had most contacts within the last month before referral. Practices are divided into CME-M group and reference group. The effect within a group is shown as an incidence rate ratio (IRR0: Reference group; IRR1: CME-M group), and comparisons between groups are shown as a ratio of incidence rate ratios. The effect on the proportion of the most attending patients within a group is shown as an odds ratio (OR0: Reference group; OR1: CME-M group), and comparisons between groups are shown as a ratio of odds ratios

*Abbreviations:* *CME-M* continuing medical education meeting, *F2F* face-to-face, *IRR* incidence rate ratio, *OR* odds ratio, *CI* confidence interval

Face-to-face (F2F) contacts include contacts with GP and patient in same room. Total contacts include face-to-face contacts, e-mails and phone contacts

^a^Adjusted for cluster, practice type, patient’s age, gender and habitual contact rate, Charlson Comorbidity Index

^b^Adjusted for cluster, patient age and gender, Charlson Comorbidity Index

^c^90^th^ centile based on all data for F2F contacts and for total contacts

Bold = significance level of *p* ≤ 0.05

### Impact of CME-M on primary care intervals

The proportion of patients who waited 14 days or more in the CME-M practices statistically significantly increased from before to after the CME-M compared to the reference practices (OR-ratio: 1.47 (95% CI: 1.06;2.03)) (Table 6). This was also seen as a tendency for other estimates although these figures were not statistically significant. When we stratified on cancer detection difficulty, no statistically significant differences were found in the primary care intervals (Table 6).

**Table 6:** The CME-M impact on patients’ primary care interval (PCI)

	Reference group *N =* 199 practices				CME-M group *N =* 139 practices				Comparison between groups	
	Before CME-M	After CME-M	Before vs. after		Before CME-M	After CME-M	Before vs. after			
			OR0^a^ (95% CI)	P			OR1^a^ (95% CI)	P	OR1/OR0^a^ (95% CI)	P
*All data, N patients*	*1,227*	*753*	*-*		*990*	*1,102*	*-*		*-*	
PCI, median days (percentiles: 75^th^, 90^th^)	1 (13, 43)	1 (12, 46)	-		1 (14, 42)	1 (17, 53)	-		-	
PCI ≥ baseline 75^th^ percentile^c^ %	25.8	24.4	0.81^2^ (0.63;1.04)	0.099	25.1	27.9	1.19^b^ (0.95;1.49)	0.135^b^	**1.47** ^b^ **(1.06;2.03)**	**0.021**
PCI ≥ baseline 90^th^ percentile^c^ %	10.1	10.6	1.12^b^ (0.79;1.57)	0.532	10.4	12.5	1.27^b^ (0.92;1.74)	0.146	1.13^b^ (0.72;1.78)	0.583
*Easier to detect, N patients*	*887*	*515*	*-*		*685*	*774*	*-*		*-*	
PCI, median days (percentiles: 75^th^, 90^th^)	0 (8, 34)	0 (7, 35)	-		0 (7, 35)	0 (9, 41)	-		-	
PCI ≥ baseline 75^th^ percentile^c^ %	26.2	23.9	0.87 (0.66;1.15)	0.345	27.2	29.7	1.18 (0.92;1.52)	0.187	1.35 (0.94;1.94)	0.101
PCI ≥ baseline 90^th^ percentile^c^ %	10.5	10.1	0.97 (0.66;1.44)	0.888	10.2	11.9	1.24 (0.87;1.77)	0.241	1.27 (0.76;2.12)	0.353
*Hard to detect, N patients*	*284*	*191*	*-*		*261*	*274*	*-*		*-*	
PCI, median days (percentiles: 75^th^, 90^th^)	7 (26.5, 71)	5 (28, 79)	-		7 (27, 58)	8 (30, 80)	-		-	
PCI ≥ baseline 75^th^ percentile^c^ %	25.0	25.7	1.12 (0.69;1.79)	0.653	25.7	30.3	1.35 (0.87;2.08)	0.180	1.21 (0.66;2.21)	0.543
PCI ≥ baseline 90^th^ percentile^c^ %	10.2	11.5	1.20 (0.62;2.34)	0.584	10.3	13.1	1.34 (0.73;2.46)	0.348	1.11 (0.48;2.59)	0.805

Primary care intervals were measured as median, 75^th^ and 90^th^ percentile and the proportion of patients who had the longest primary care interval before and after the CME-M. The analyses are also stratified on cancer detection difficulty. Practices are divided into CME-M group and reference group. An effect within a group is shown as an odds ratio (OR0: Reference group; OR1: CME-M group). Comparisons between groups are shown as a ratio of odds ratios

The suspected cancer types were divided into two groups based on cancer detection difficulty: 1) easier to detect included suspected cancer in kidney, bladder, breast, head and neck, female genitalia, nevus (melanoma), penis, testis and gastrointestinal and 2) harder to detect included suspected cancer in pancreas, liver and gall bladder, brain, lymph node and bone marrow, lung, prostate, connective tissue including fat, muscle and bones, and non-specific serious symptoms

*Abbreviations:* *PCI* primary care interval, *CME-M* continuing medical education meeting, *OR* Odds ratio, *CI* confidence interval

^a^Adjusted for cluster, practice type, patient gender, age and comorbidity

^b^In addition, adjusted for cancer detection difficulty

^c^75^th^ and 90^th^ percentiles of baseline measurements (before the CME-M) were used for each group as basis

Bold = significance level of *p ≤* 0.05

### Sensitivity analyses

When we excluded patients urgently referred within one or two months after the CME-M date, the CME-M effect on *urgent referral rates* and *prior contacts* did not differ considerably from the main results (Additional file 2).

## Discussion

### Main findings

Forty percent of general practices participated in the cancer diagnostic CME-M. Increased use of urgent referrals was found in the CME-M group from before to after the CME-M. However, this increase was only small in absolute numbers and was not statistically significantly different from the figures for the reference group. Compared with the reference group, the CME-M seemed to reduce the number of total contacts with general practice in the CME-M group during the last month before the urgent referral. Furthermore, the proportion of patients with more than five contacts in total during the last month decreased in the CME-M group; together this indicates a lower referral threshold.

The CME-M was associated with an increase in the reported primary care intervals.

### Strength and limitations

The before-after design allowed us to perform a robust evaluation with corrections for baseline measures of a natural experiment. We controlled for calendar time by comparing CME-M group and reference group and by including the stepwise enrolment of GPs in the CME-M into the modelling of data, which diminished the influence of increasing cancer-related knowledge over time.

Another strength was the well-defined and well-described study population and the valid identification of the individuals listed with the studied practices. Additionally, we were able to link selected information on all included individuals to relevant register data at the individual level.

The main limitation of this study was the natural selection to participate in the CME-M. Participating practices and GPs differed from non-participating practices and GPs, particularly single-handed practices differed on the use of urgent referrals (Table 4). This indicates that CME-M participating practices cannot be directly compared to non-participating practices. The CME-M practices had a higher use of urgent referrals among 1000 patients at baseline; this indicates that their potential for improvement was lower than for the entire study base, which may have underestimated the effect of the CME-M. However, the true direction of such bias is difficult to establish.

The use of the referral algorithm allowed us to base the identification of urgently referred patients on register data [31]. We have no reason to believe that patients from CME-M practices had different chances of being identified by the algorithm than patients from reference practices. On the contrary, the patient population used for the evaluation of the CME-M effect on the primary care interval required that the referring GP completed the one-page form about the urgently referred patient. The CME-M practices were more likely to complete one-page forms (Table 2), whereas the reference practices lost compliance over time. We do not know whether this was a differentiated non-response (e.g. in cases with long primary care intervals), but it could be the case as also indicated by the paradoxical result. However, the proportion of patients in the “harder to detect” group of referrals actually increased in the reference group after the CME-M [39].

To investigate the reasons for the observed increase in the primary care interval combined with an indication of lowered referral threshold, we performed sensitivity analyses. These did not point towards a delayed effect of the CME-M or a “cleaning up process” of patients who had recently been seen in general practice because of unspecific symptoms. The most likely explanation of our finding is a change in the awareness of unspecific symptoms among GPs in CME-M practices, which could have led the GPs to report an earlier date of first symptom presentation. Thus, the GPs may have gained a more realistic idea about the usual time intervals in general practice for patients suspected of cancer.

### Comparison with other studies and clinical implications

To our knowledge, this study is among the first to evaluate a CME-M on GPs’ use and timing of urgent cancer referrals. In accordance with our findings, an English before-after study found that the use of RATs for lung and colorectal cancer in general practice was associated with an increased number of urgent referrals during a 6-month period [43]. The reason for this could be that the GPs’ awareness of potential cancer symptoms and attitude towards urgent cancer referral changed through the CME-M. Similar results have also been found in qualitative studies about the use of RATs [44, 45] and in our previous analysis of the impact of the CME-M on GP knowledge, attitude and intentions [18].

Our intervention was developed on the basis of theoretical frameworks and reviews of empirical results to ensure optimal likelihood of positive effect [44–47]. The CME-M consisted of a variety of elements [18], and we could not validly identify the most effective parts of the CME-M; this calls for further research in changing the GPs’ behaviour [46].

In our study, the non-participating practices were more often single-handed, the GPs were more often males, and their practice population was larger, more deprived and with more male patients than in participating practices. Similar findings were also reported in a comprehensive quality development project on chronic diseases conducted in the Central Denmark Region in 2010 [47]. These findings may result from differences in the GPs’ possibility to participate (e.g. time constraints) [48]. However, it may also be explained by the theories of varying readiness for change of behaviour [49]; some GPs belong to a group known to be susceptible for new knowledge (innovators, early adopters and early majority), whereas other GPs are known to be sceptical (late majority and laggards). One way to change the pattern of non-participation could perhaps be to develop specific CME initiatives to better fit the GPs’ preferences for education and individual learning style [50].

## Conclusion

We found a statistically significant reduction in the number of total contacts with general practice within the month preceding an urgent referral and an increase in the reported primary care intervals from before to after in the CME-M group compared to the reference group. A CME-M may contribute to changing the threshold for referring the patients and the GPs’ understanding of the primary care interval.

## Additional files

Additional file 1: One page forms used for collection of patient information. (PDF 560 kb)

Additional file 2: Tables on sensitivity analyses. Additional file 2 includes Additional Tables I-V. Additional **Table I**: The CME-M impact on patients’ prior contacts with general practice stratified on practice type. Additional **Table II**: The CME-M impact on patients’ primary care interval stratified on both practice type and cancer detection difficulty. Additional **Table III**: The CME-M impact on patients’ prior contacts with general practice with exclusion of patients referred within 30 days following the CME-M-date. Additional **Table IV**: The CME-M impact on patients’ primary care interval with exclusion of patients referred within 30 days following the CME-M-date. Additional **Table V**: The CME-M impact on patients’ primary care interval with exclusion of patients referred within 61 days following the CME-M-date. (DOCX 68 kb)

## Abbreviations

CME-M: Continuing medical education meeting
CPR: Civil personal registration
DADI: Danish deprivation index
F2F: Face to face
GP: General practitioner
IRR: Incidence rate ratio
OR: Odds ratio
RATs: Risk assessment tools
