# Supplementary material for: Impact of a continuing medical education meeting on the use and timing of urgent cancer referrals among general practitioners - a before-after study
Source: BMC Fam Pract. 2017 Mar 21;18:44. doi: 10.1186/s12875-017-0607-3 (PMC5361715; doi:10.1186/s12875-017-0607-3)
Supplement: Supplementary file 1 — One page forms used for collection of patient information. (PDF 560 kb) [file 12875_2017_607_MOESM1_ESM.pdf]

Additional file 1a: One-page form. The GP was requested to complete a form each time the GP referred a patient to a cancer fast-track pathway

CaP
Research Centre for
Cancer Diagnosis in Primary Care

AARHUS UNIVERSITET

Please complete this form each time you refer a patient to a cancer fast-track pathway

Insert the date of referral of your patient to a cancer fast-track pathway:

day month year

☐☐☐☐☐☐

Enter your patient´s personal identification number:

day month year xxxx

☐☐☐☐☐☐ ☐☐☐☐

Enter the type of cancer fast-track pathway the patient is referred to:

______________________________ ☐☐

Enter your degree of certainty in your choice of which cancer fast-track pathway you referred the patient to:

☐ Totally certain

☐ Fairly certain

☐ Little uncertain

☐ Totally uncertain

Please elaborate______________________________ ☐☐

On which symptoms did you refer the patients?

Symptom 1: ______________________________ ☐☐☐

Symptom 2: ______________________________ ☐☐☐

Symptom 3: ______________________________ ☐☐☐

Insert the date of the day the patient first presented symptoms in general practice which according to your present knowledge raised your suspicion that the patient could have cancer:

day month year

☐☐☐☐☐☐

What is the risk for this patient having cancer?

(0 % = no risk, 100 % = absolutely certain)

☐☐☐ ☐ I don´t know

Additional file 1b: One-page reminder form, used if we obtained information from register data about a patient investigated in a cancer fast-track pathway and we have not yet received a form.

CaP
Research Centre for
Cancer Diagnosis in Primary Care

AARHUS UNIVERSITET

Form on a patient investigated in a cancer fast-track pathway

From register data, we have been informed that a patient with the following personal identification number listed at your general practice has been investigated in a cancer fast-track pathway. We kindly request the referring general practitioner to complete this form.

1	1	1	1	1	1		1	1	1	1

Enter your full name or your project ID-number (this number appears on your pad):

____ ☐☐☐☐☐

Referral options:

- ☐ I have referred the patient to a cancer fast-track pathway
- ☐ I have referred the patient in the usual way to a hospital on cancer suspicion, but not directly to a cancer fast-track pathway
- ☐ I have referred the patient in the usual way to a hospital and did not suspect cancer
- ☐ The patient was admitted to hospital by emergency
- ☐ Suspicion of cancer was based on screening
- ☐ I have not been involved

Other ____ ☐☐

Enter the degree of certainty in your choice of cancer fast-track pathway:

- ☐ Totally certain
- ☐ Fairly certain
- ☐ Little uncertain
- ☐ Totaly uncertain

Please elaborate____ ☐☐

Insert the date of the day the patient first presented symptoms in general practice which according to your present knowledge raised your suspicion that the patient could have cancer:

day month year

What is the risk that this patient has cancer?

(0 % = no risk, 100 % = absolutely high risk)

☐☐☐ ☐ I don´t know

Insert the date of referral of your patient to a cancer fast-track pathway:

day month year

☐☐☐☐☐☐

On which symptoms did you refer the patient?

Symptom 1: ____ ☐☐☐

Symptom 2: ____ ☐☐☐

Symptom 3: ____ ☐☐☐
