# Supplementary material for: Impact of a continuing medical education meeting on the use and timing of urgent cancer referrals among general practitioners - a before-after study
Source: BMC Fam Pract. 2017 Mar 21;18:44. doi: 10.1186/s12875-017-0607-3 (PMC5361715; doi:10.1186/s12875-017-0607-3)
Supplement: Supplementary file 2 — Tables on sensitivity analyses. Additional file 2 includes Additional Tables I-V. Additional Table I: The CME-M impact on patients’ prior contacts with general practice stratified on practice type. Additional Table II: The CME-M impact on patients’ primary care interval stratified on both practice type and cancer detection difficulty. Additional Table III: The CME-M impact on patients’ prior contacts with general practice with exclusion of patients referred within 30 days following the CME-M-date. Additional Table IV: The CME-M impact on patients’ primary care interval with exclusion of patients referred within 30 days following the CME-M-date. Additional Table V: The CME-M impact on patients’ primary care interval with exclusion of patients referred within 61 days following the CME-M-date. (DOCX 68 kb) [file 12875_2017_607_MOESM2_ESM.docx]

| **Additional table I** The CME-M impact on patients´ prior contacts with general practice *stratified on practice type.* | | | | | | | |
| --- | --- | --- | --- | --- | --- | --- | --- |
|  | Reference group | | | CME-M group | | | Comparison btw. groups |
|  | Before  CME-M | After  CME-M | Before vs. after | Before  CME-M | After  CME-M | Before vs. after |  |
| Prior contacts |  |  | IRR0 (95% CI)^1^ |  |  | IRR1 (95% CI)^1^ | IRR1/IRR0 (95% CI)^1^ |
| Single-handed practices | 144 | |  | 42 | |  |  |
| N patients | 2,866 | 2,906 |  | 942 | 957 |  |  |
| F2F contacts (mean) |  |  |  |  |  |  |  |
| 0-1 month | 1.90 | 1.92 | 1.01(0.98;1.05) | 1.88 | 1.88 | 1.00(0.94;1.05) | 0.98(0.92;1.05) |
| 0-3 months | 3.24 | 3.20 | 1.00(0.97;1.03) | 3.15 | 3.15 | 1.00(0.95;1.06) | 1.00(0.94;1.07) |
| 4-6 months | 1.87 | 1.76 | 0.99(0.93;1.04) | 1.74 | 1.82 | 1.07(0.98;1.18) | 1.09(0.98;1.22) |
| Total contacts (mean) |  |  |  |  |  |  |  |
| 0-1 month | 2.81 | 2.87 | 1.03(0.996;1.06) | 3.09 | 3.07 | 0.99(0.94;1.04) | 0.96(0.90;1.02) |
| 0-3 months | 5.15 | 5.22 | 1.02(0.99;1.05) | 5.60 | 5.61 | 1.00(0.95;1.06) | 0.98(0.92;1.04) |
| 4-6 months | 3.32 | 3.21 | 0.99(0.95;1.04)^2^ | 3.51 | 3.47 | 1.01(0.93;1.09)^2^ | 1.01(0.92;1.11)^2^ |
| Most contacting patients in the month preceding referral |  |  | OR0 (95% CI)^1^ |  |  | OR1 (95% CI)^1^ | OR1/OR0 (95% CI)^1^ |
| F2F-contacts ≥ 4^3^ % | 10.4 | 10.9 | 1.11(0.93;1.31) | 10.3 | 10.1 | 0.99(0.73;1.35) | 0.90(0.63;1.27) |
| Total contacts ≥ 6^3^ % | 9.0 | 9.8 | 1.14(0.95;1.38) | 11.8 | 12.4 | 1.05(0.78;1.41) | 0.92(0.65;1.31) |
| Partnership practices | 96 | |  | 102 | |  |  |
| N patients | 4,793 | 4,825 |  | 6,179 | 6,460 |  |  |
| F2F contacts (mean) |  |  |  |  |  |  |  |
| 0-1 month | 1.92 | 1.88 | 0.98(0.96;1.01) | 1.93 | 1.90 | 0.98(0.96;1.01) | 1.00(0.97;1.04) |
| 0-3 months | 3.24 | 3.21 | 1.00(0.97;1.02) | 3.24 | 3.17 | 1.00(0.98;1.02) | 1.00(0.97;1.04) |
| 4-6 months | 1.79 | 1.83 | 1.03 (0.98;1.07) | 1.85 | 1.84 | 0.99(0.95;1.02) | 0.96(0.91;1.02) |
| Total contacts (mean) |  |  |  |  |  |  |  |
| 0-1 month | 2.87 | 2.87 | 1.00(0.98;1.03) | 2.93 | 2.84 | **0.98(0.96;0.9999)** | 0.97(0.94;1.01) |
| 0-3 months | 5.18 | 5.21 | 1.01(0.98;1.03) | 5.27 | 5.13 | 0.99(0.97;1.01) | 0.98(0.95;1.02) |
| 4-6 months | 3.17 | 3.26 | 1.01(0.97;1.06)^2^ | 3.23 | 3.17 | 0.97(0.94;1.01)^2^ | 0.96(0.91;1.02)^2^ |
| Most contacting patients in the month preceding referral |  |  |  |  |  |  |  |
| F2F-contacts ≥ 4^3^ % | 10.4 | 10.1 | 0.97(0.84;1.11) | 10.9 | 10.9 | 0.99(0.88;1.12) | 1.03(0.86;1.23) |
| Total contacts ≥ 5^3^ % | 17.4 | 17.2 | 1.00(0.89;1.11) | 18.3 | 17.0 | 0.92(0.84;1.02) | 0.93(0.80;1.07) |

Prior contacts are shown as face-to-face and total contacts for three time periods: 0-1, 0-3 and 4-6 months and as proportions of patients who had most contacts within the last month before referral. Practices are divided into CME-M group and reference group. The effect within a group is shown as an incidence rate ratio (IRR0: Reference group; IRR1: CME-M group), and comparisons between groups are shown as a ratio of incidence rate ratios. The effect on the proportion of the most attending patients within a group is shown as an odds ratio (OR0: Reference group; OR1: CME-M group), and comparisons between groups are shown as a ratio of odds ratios.

Abbreviations: CME-M, continuing medical education meeting; F2F, face-to-face; IRR, incidence rate ratio; OR, Odds ratio; CI, confidence interval; btw, between

Face-to-face contacts included contacts with GP and patient in same room. Total contacts include face-to-face contacts, e-mails and phone contacts.

^1^Adjusted for cluster, patient´s age, gender and habitual monthly contact rate, Charlson Comorbidity Index

^2^Only adjusted for cluster, patient´s age and gender, Charlson Comorbidity Index

^3^90^th^ centile based on all data was 4 for F2F contacts for both practice type, and for total contacts it was 5 for partnerships practices but 6 for single-handed practices.

Bold=significance level of p≤0.05.

| **Additional table II** The CME-M impact on patients´ primary care interval (PCI) *stratified on practice type and cancer detection difficulty.* | | | | | | | | |
| --- | --- | --- | --- | --- | --- | --- | --- | --- |
|  | Reference group | | |  | CME-M group | | | Comparison between groups |
|  | Before  CME-M | After  CME-M | Before vs. after  OR0 (95% CI)^2^ | | Before  CME-M | After  CME-M | Before vs. after  OR1 (95% CI)^2^ | OR1/OR0  (95% CI)^2^ |
| Single-handed practices | 106 |  |  | | 37 |  |  |  |
| All data, N patients | 501 | 295 | - | | 170 | 149 | - | - |
| PCI, median days (percentiles: 75^th^, 90^th^) | 1(13, 43) | 1(13, 45) | - | | 0.5(12, 36.5) | 1(18, 71) | - | - |
| PCI ≥ baseline 75^th^ percentile^1^ % | 25.7 | 25.4 | 0.82(0.54;1.25)^3^ | | 25.3 | 31.5 | 1.45(0.78;2.70)^3^ | 1.77(0.86;3.67)^3^ |
| PCI ≥ baseline 90^th^ percentile^1^ % | 10.4 | 10.5 | 1.08(0.60;1.92)^3^ | | 10.0 | 15.4 | 1.36(0.59;3.14)^3^ | 1.26(0.48;3.35)^3^ |
| Easier to detect, N patients | 361 | 201 | - | | 114 | 109 | - | - |
| PCI, median days (percentiles: 75^th^, 90^th^) | 0(9, 26) | 0(6, 33) | - | | 0(7, 32) | 0(7, 39) | - | -- |
| PCI ≥ baseline 75^th^ percentile^1^ % | 25.2 | 20.9 | 0.71(0.44;1.15) | | 27.2 | 27.5 | 1.03(0.50;2.12) | 1.46(0.63;3.36) |
| PCI ≥ baseline 90^th^ percentile^1^ % | 11.1 | 12.9 | 1.16(0.63;2.14) | | 10.5 | 13.8 | 1.44(0.54;3.84) | 1.24(0.40;3.77) |
| Hard to detect, N patients | 116 | 73 | - | | 51 | 32 | - | - |
| PCI, median days (percentiles: 75^th^, 90^th^) | 8(29.5, 146) | 4(22, 79) | - | | 5(18, 42) | 12.5(30.5, 168) | - | - |
| PCI ≥ baseline 75^th^ percentile^1^ % | 25.0 | 19.2 | 0.97(0.37;2.55) | | 25.5 | 40.6 | 2.19(0.71;6.75) | 2.26(0.55;9.32) |
| PCI ≥ baseline 90^th^ percentile^1^ % | 10.3 | 6.8 | 0.70(0.17;2.90)^4^ | | 13.7 | 21.9 | 1.19(0.26;5.39)^4^ | 1.70(0.22;12.9)^4^ |
| Partnership practices | 93 | |  | | 102 | |  |  |
| All data, N patients | 726 | 458 | - | | 820 | 953 | - | - |
| PCI, median days (percentiles: 75^th^, 90^th^) | 1(13, 42) | 1(11, 46) | - | | 1(14, 44) | 1(17, 53) | - | - |
| PCI ≥ baseline 75^th^ percentile^1^ % | 25.9 | 23.8 | 0.81(0.59;1.10)^3^ | | 25.6 | 27.6 | 1.15(0.90;1.47)^3^ | 1.42(0.97;2.09)^3^ |
| PCI ≥ baseline 90^th^ percentile^1^ % | 10.3 | 11.8 | 1.30(0.86;1.99)^3^ | | 10.1 | 11.4 | 1.24(0.87;1.76)^3^ | 0.95(0.56;1.62)^3^ |
| Easier to detect, N patients | 526 | 314 | - | | 571 | 665 | - | - |
| PCI, median days (percentiles: 75^th^, 90^th^) | 0(8, 36) | 0(7, 35) | - | | 0(7, 35) | 0(9, 42) | - | - |
| PCI ≥ baseline 75^th^ percentile^1^ % | 25.5 | 24.5 | 0.99(0.70;1.41) | | 27.1 | 30.1 | 1.22(0.94;1.60) | 1.23(0.81;1.89) |
| PCI ≥ baseline 90^th^ percentile^1^ % | 10.1 | 9.9 | 1.04(0.63;1.74) | | 10.5 | 11.9 | 1.23(0.84;1.81) | 1.18(0.63;2.18) |
| Hard to detect, N patients | 168 | 118 | - | | 210 | 242 | - | - |
| PCI, median days (percentiles: 75^th^, 90^th^) | 7(23, 60) | 6.5(33, 84) | - | | 10(28, 63.5) | 8(30, 70) | - | - |
| PCI ≥ baseline 75^th^ percentile^1^ % | 26.8 | 30.5 | 1.26(0.71;2.23) | | 26.2 | 29.3 | 1.21(0.76;1.95) | 0.97(0.47;1.96) |
| PCI ≥ baseline 90^th^ percentile^1^ % | 10.1 | 12.7 | 1.21(0.55;2.67)^4^ | | 10.0 | 12.0 | 1.30(0.66;2.56)^4^ | 1.08(0.40;2.91)^4^ |

Primary care intervals (PCI) were measured as median, 75^th^ and 90^th^ percentile and the proportion of patients who had the longest primary care interval before and after the CME-M. Practices are divided into CME-M group and reference group. An effect within a group is shown as an odds ratio (OR0: Reference group; OR1: CME-M-group). Comparisons between groups are shown as a ratio of odds ratios.

The suspected cancer types were divided into two groups based on cancer detection difficulty: 1) easier to detect included suspected cancer in kidney, bladder, breast, head and neck, female genitalia, nevus (melanoma), penis, testis and gastrointestinal, and 2) harder to detect included suspected cancer in pancreas, liver and gall bladder, brain, lymph node and bone marrow, lung, prostate, connective tissue including fat, muscle and bones, and suspicion based on unspecific, serious symptoms.

Abbreviations: PCI, primary care interval; CME-M, continuing medical education meeting; OR, Odds ratio; CI, confidence interval

^1^75^th^ and 90^th^ percentiles of baseline measurements (Before CME-M) were used for each group as basis. ^2^Adjusted for cluster, patients´ gender, age and comorbidity. ^3^In addition, adjusted for cancer detection difficulty. ^4^Only adjusted for cluster, patients´ gender and age.

| **Additional table III** The CME-M impact on patients´ prior contacts with general practice. *Patients referred within 30 days (d) following the CME-M-date were excluded.* | | | | | | | |
| --- | --- | --- | --- | --- | --- | --- | --- |
|  | Reference group  N= 240 practices | | | CME-M group  N= 144 practices | | | Comparison between groups |
| N patients | Before  CME-M  7659 | After  CME-M +30d  6360 | Before vs. after | Before  CME-M  7121 | After  CME-M +30d  6037 | Before vs. after |  |
| Prior contacts |  |  | IRR0 (95% CI)^1^ |  |  | IRR1 (95% CI)^1^ | IRR1/IRR0 (95% CI)^1^ |
| F2F contacts |  |  |  |  |  |  |  |
| 0-1 month, mean | 1.91 | 1.89 | 0.99(0.97;1.01) | 1.92 | 1.88 | 0.98(0.96;1.00) | 0.99(0.95;1.02) |
| 0-3 months, mean | 3.24 | 3.20 | 0.98(0.96;1.01) | 3.23 | 3.19 | 0.98(0.96;1.01) | 1.00(0.97;1.03) |
| 4-6 months, mean | 1.82 | 1.82 | 1.02(0.98;1.05) | 1.84 | 1.82 | 0.99(0.96;1.03) | 0.98(0.93;1.03) |
| Total contact |  |  |  |  |  |  |  |
| 0-1 month, mean | 2.84 | 2.87 | 1.01(0.99;1.03) | 2.95 | 2.83 | **0.97(0.95;0.99)** | **0.96(0.93;0.99)** |
| 0-3 months, mean | 5.17 | 5.21 | 1.01(0.99;1.03) | 5.31 | 5.12 | 0.98(0.96;1.00) | 0.97(0.94;1.00) |
| 4-6 months, mean | 3.23 | 3.26 | 1.01(0.97;1.04)^2^ | 3.27 | 3.17 | 0.96(0.93;1.00)^2^ | 0.96(0.91;1.01)^2^ |
| Most contacting patients in the month preceding referral |  |  | OR0 (95% CI)^1^ |  |  | OR1 (95% CI)^1^ | OR1/OR0 (95% CI)^1^ |
| F2F contacts ≥ 4^3^ (%) | 10.4 | 10.4 | 1.02(0.91;1.14) | 10.8 | 10.6 | 0.97(0.87;1.09) | 0.96(0.81;1.12) |
| Total contacts ≥ 5^3^ (%) | 16.9 | 17.2 | 1.03(0.94;1.13) | 18.5 | 17.1 | 0.92(0.83;1.01) | 0.89(0.78;1.02) |

Prior contacts are shown as face-to-face and total contacts for three time periods: 0-1, 0-3 and 4-6 months and as proportions of patients who had most contacts within the last month before referral. Practices are divided into CME-M group and reference group. The effect within a group is shown as an incidence rate ratio (IRR0: Reference group; IRR1: CME-M group), and comparisons between groups are shown as a ratio of incidence rate ratios. The effect on the proportion of the most attending patients within a group is shown as an odds ratio (OR0: Reference group; OR1: CME-M group), and comparisons between groups are shown as a ratio of odds ratios.

Abbreviations: CME-M, continuing medical education meeting; F2F, face-to-face; IRR, incidence rate ratio; OR, Odds ratio; CI, confidence interval.

Face-to-face contacts included contacts with GP and patient in same room. Total contacts include face-to-face contacts, e-mails and phone contacts.

^1^Adjusted for cluster, patient´s age, gender and habitual monthly contact rate, Charlson Comorbidity Index

^2^Only adjusted for cluster, patient´s age and gender, Charlson Comorbidity Index

^3^90^th^ centile based on all data was 4 for F2F contacts and 5 for total contacts.

Bold=significance level of p≤0.05.

| **Additional table IV** The CME-M impact on patients´ primary care interval (PCI). *Patients referred within 30 days (d) following the CME-M were excluded.* | | | | | | | | |
| --- | --- | --- | --- | --- | --- | --- | --- | --- |
|  | Reference group  N= 197 practices | | |  | CME-M group  N= 139 practices | | | Comparison between groups |
|  | Before  CME-M | After  CME-M +30d | Before vs. after  OR0 (95% CI)^2^ | | Before  CME-M | After  CME-M +30d | Before vs. after  OR1 (95% CI)^2^ | OR1/OR0 (95% CI)^2^ |
| All data, N patients | *1227* | *503* | *-* | | *990* | *791* | *-* | *-* |
| PCI, median days (percentiles: 75^th^, 90^th^) | 1(13,43) | 1(12,45) | - | | 1(14,42) | 1(15,54) | - | - |
| PCI ≥ baseline 75^th^ percentile^1^ % | 25.8 | 24.9 | 0.75(0.55;1.01)^3^ | | 25.1 | 26.9 | 1.09(0.84;1.41)^3^ | **1.46(1.00;2.12)^3^** |
| PCI ≥ baseline 90^th^ percentile^1^ % | 10.1 | 10.1 | 1.07(0.71;1.62)^3^ | | 10.4 | 12.6 | 1.34(0.93;1.92)^3^ | 1.25(0.75;2.08)^3^ |
| *Easier to detect, N patients* | *887* | *324* | *-* | | *685* | *556* | *-* | *-* |
| PCI, median days (percentiles: 75^th^, 90^th^) | 0(8,34) | 0(7,28) | - | | 0(7,35) | 0(7,43) | - | - |
| PCI ≥ baseline 75^th^ percentile^1^ % | 26.2 | 23.5 | 0.80(0.57;1.13) | | 27.2 | 27.2 | 0.98(0.74;1.31) | 1.23(0.80;1.87) |
| PCI ≥ baseline 90^th^ percentile^1^ % | 10.5 | 8.6 | 0.82(0.50;1.36) | | 10.2 | 12.4 | 1.28(0.86;1.90) | 1.56(0.86;2.83) |
| *Hard to detect, N patients* | *284* | *145* | *-* | | *261* | *199* | *-* | *-* |
| PCI, median days (percentiles: 75^th^, 90^th^) | 7(26.5,71) | 5(28,75) | - | | 7(27,58) | 8(31,67) | - | - |
| PCI ≥ baseline 75^th^ percentile^1^ % | 25.0 | 25.5 | 1.17(0.69;2.00) | | 25.7 | 29.6 | 1.43(0.86;2.36) | 1.22(0.63;2.36) |
| PCI ≥ baseline 90^th^ percentile^1^ % | 10.2 | 11.0 | 1.09(0.52;2.29)^4^ | | 10.3 | 11.1 | 1.05(0.52;2.14)^4^ | 0.97(0.39;2.41)^4^ |

Primary care intervals (PCI) were measured as median, 75^th^ and 90^th^ percentile and the proportion of patients who had the longest primary care interval before and after the CME-M. The analyses are also stratified on cancer detection difficulty. Practices are divided into CME-M group and reference group. An effect within a group is shown as an odds ratio (OR0: Reference group; OR1: CME-M group). Comparisons between groups are shown as a ratio of odds ratios.

Abbreviations: PCI, primary care interval; CME-M, continuing medical education meeting; OR, Odds ratio; CI, confidence interval; d, days.

^1^75^th^ and 90^th^ percentiles of baseline measurements (Before CME-M) were used for each group as basis. ^2^Adjusted for a cluster, patients´ gender, age and comorbidity. ^3^In addition, adjusted for cancer detection difficulty. ^4^Only adjusted for cluster, patients´ gender and age.

The suspected cancer types were divided into two groups based on cancer detection difficulty: 1) easier to detect included suspected cancer in kidney, bladder, breast, head and neck, female genitalia, nevus (melanoma), penis, testis and gastrointestinal, and 2) harder to detect included suspected cancer in pancreas, liver and gall bladder, brain, lymph node and bone marrow, lung, prostate, connective tissue including fat, muscle and bones, and suspicion based on unspecific, serious symptoms.

Bold=significance level of p≤0.05.

| **Additional table V** The CME-M impact on patients´ primary care interval (PCI). *Patients referred within 61 days (d) following the CME-M were excluded.* | | | | | | | | |
| --- | --- | --- | --- | --- | --- | --- | --- | --- |
|  | Reference group  N= 191 practices | | |  | CME-M group  N= 139 practices | | | Comparison between groups |
|  | Before  CME-M | After  CME-M +61d | Before vs. after  OR0 (95% CI)^2^ | | Before  CME-M | After  CME-M +61d | Before vs. after  OR1 (95% CI)^2^ | OR1/OR0 (95% CI)^2^ |
| All data, N patients | *1227* | *280* | *-* | | *990* | *532* | *-* | *-* |
| PCI, median days (percentiles: 75^th^, 90^th^) | 1(13,43) | 1(11;35.5) | - | | 1(14,42) | 1(14.5,55) | - | - |
| PCI ≥ baseline 75^th^ percentile^1^ % | 25.8 | 23.2 | 0.74(0.50;1.09)^3^ | | 25.1 | 26.3 | 1.13(0.82;1.54)^3^ | 1.52(0.97;2.39)^3^ |
| PCI ≥ baseline 90^th^ percentile^1^ % | 10.1 | 8.2 | 0.90(0.51,1.58)^3^ | | 10.4 | 11.7 | 1.22(0.78;1.92)^3^ | 1.37(0.72,2.61)^3^ |
| *Easier to detect, N patients* | *887* | *175* | *-* | | *685* | *370* | *-* | *-* |
| PCI, median days (percentiles: 75^th^, 90^th^) | 0(8,34) | 0(6,21) | - | | 0(7,35) | 0(7,38) | - | - |
| PCI ≥ baseline 75^th^ percentile^1^ % | 26.2 | 20.6 | 0.64(0.40,1.03) | | 27.2 | 25.1 | 0.89(0.63;1.26) | 1.38(0.81,2.36) |
| PCI ≥ baseline 90^th^ percentile^1^ % | 10.5 | 7.4 | 0.72(0.36;1.45) | | 10.2 | 11.1 | 1.19(0.73;1.94) | 1.65(0.77,3.54) |
| *Hard to detect, N patients* | *284* | *94* | *-* | | *261* | *141* | *-* | *-* |
| PCI, median days (percentiles: 75^th^, 90^th^) | 7(26.5,71) | 5(22,66) | - | | 7(27,58) | 8(30,67) | - | - |
| PCI ≥ baseline 75^th^ percentile^1^ % | 25.0 | 22.3 | 1.16(0.59;2.25) | | 25.7 | 28.4 | 1.45(0.80;2.61) | 1.25(0.58;2.68) |
| PCI ≥ baseline 90^th^ percentile^1^ % | 10.2 | 11.0 | 0.97(0.36;2.59)^4^ | | 10.3 | 11.1 | 1.16(0.49;2.78)^4^ | 1.20(0.39;3.72)^4^ |

Primary care intervals (PCI) were measured as median, 75^th^ and 90^th^ percentile and the proportion of patients who had the longest primary care interval before and after the CME-M. The analyses are also stratified on cancer detection difficulty. Practices are divided into CME-M group and reference group. An effect within a group is shown as an odds ratio (OR0: Reference group; OR1: CME-M group). Comparisons between groups are shown as a ratio of odds ratios.

Abbreviations: PCI, primary care interval; CME-M, continuing medical education meeting; OR, Odds ratio; CI, confidence interval; d, days.

^1^75^th^ and 90^th^ percentiles of baseline measurements (Before CME) were used for each group as basis. ^2^Adjusted for a cluster, patients´ gender, age and comorbidity. ^3^In addition, adjusted for cancer detection difficulty. ^4^Only adjusted for cluster, patients´ gender and age.

The suspected cancer types were divided into two groups based on cancer detection difficulty: 1) easier to detect included suspected cancer in kidney, bladder, breast, head and neck, female genitalia, nevus (melanoma), penis, testis and gastrointestinal, and 2) harder to detect included suspected cancer in pancreas, liver and gall bladder, brain, lymph node and bone marrow, lung, prostate, connective tissue including fat, muscle and bones, and suspicion based on unspecific, serious symptoms.
